# The influence of mobile health intervention on the rate of prenatal diagnosis and pregnancy outcomes among pregnant women with high-risk prenatal screening results: protocol for a randomized controlled trial

**DOI:** 10.3389/fpsyt.2025.1582368

**Published:** 2025-06-04

**Authors:** Jie Wang, Jing Wang, Yang Jiang, Yaxian Liu, Rantong Bao, Hong Wang, Yan Huang, Jin An, Xiaohua Wang, Fei Wang

**Affiliations:** ^1^ Department of Genetics, Inner Mongolia Maternity and Child Health Care Hospital, Hohhot, China; ^2^ School of Public Health, Peking University, Beijing, China; ^3^ Jitang College, North China University of Science and Technology, Tangshan, Hebei, China; ^4^ Department of Quality Management, Affiliated Hospital of Inner Mongolia Medical University, Hohhot, Inner Mongolia, China; ^5^ Department of Obstetrics, Inner Mongolia Maternity and Child Health Care Hospital, Hohhot, China; ^6^ Shandong Key Laboratory of Reproductive Research and Birth Defect Prevention, Gynecology Department, Shandong Provincial Hospital Affiliated to Shandong First Medical University, Jinan, Shandong, China; ^7^ Gynecology Department, Inner Mongolia Maternity and Child Health Care Hospital, Hohhot, Inner Mongolia Autonomous Region, Hohhot, China

**Keywords:** mobile health, behavior change wheel, family health theory, prenatal diagnosis, high-risk pregnancy, pregnancy outcomes

## Abstract

**Background:**

Prenatal diagnostics is a crucial process for ensuring the health of both pregnant women and their fetuses. However, the participation rate of high-risk pregnant women in prenatal diagnostics is often influenced by various factors, including anxiety, depression, and lack of family support. In recent years, mobile health (mHealth) interventions have become an important tool in improving maternal health management, especially in terms of behavior change. The Behavior Change Wheel (BCW) theory and the Family Health Theory (FHT) have been applied in various health interventions, but there are limited studies focusing on prenatal diagnostics.

**Objective:**

The study aims to evaluate the impact of a mobile health intervention based on the Behavior Change Wheel theory and Family Health Theory on prenatal diagnostic participation rates and pregnancy outcomes in high-risk pregnant women, while exploring the role of family member involvement in improving maternal psychological health and pregnancy outcomes.

**Methods:**

The study outlines a single-blind, interventional, randomized controlled trial conducted at the Inner Mongolia Maternity and Child Health Care Hospital. A total of 58 high-risk pregnant women will be included and randomly assigned to either the intervention group (29 participants) or the standard care group (29 participants). The intervention group will receive a 12-week mHealth intervention via the WeChat platform, including health education, emotional support, behavioral feedback, and family member participation. The standard care group will receive standard prenatal care. Primary outcomes include prenatal diagnostic needs and pregnancy outcomes, while secondary outcomes include health knowledge, anxiety and depression levels, decision conflict, and other factors. This study uses IBM SPSS Statistics 24.0 for data analysis, employing descriptive statistics, normality tests, Mann–Whitney U, Wilcoxon, and chi-square tests.

**Discussion:**

The study proposes that a mobile health intervention based on the Behavior Change Wheel theory and Family Health Theory may effectively increase prenatal diagnostic participation and improve the psychological health and pregnancy outcomes of high-risk pregnant women. The active participation and emotional support of family members are expected to be key components in achieving these improvements. This research provides new insights and evidence for the application of mHealth interventions in prenatal screening.

## 1 Introduction

Birth defects refer to abnormalities in the structure, function, or metabolism of a baby that result from genetic, environmental, or their interaction, occurring before birth (1). As one of the leading causes of disability and death in children, birth defects have profound physical and psychological effects on the affected children and their families, while also imposing a significant socio-economic burden (2–4). According to the latest epidemiological data, the incidence of birth defects in China is approximately 6%, with around 900,000 new cases reported annually, and this rate continues to rise (5, 6). A meta-analysis study by Blencowe et al. reported that the global birth defect rate in live births is approximately 2%-3%, equating to about 8 million new cases of birth defects each year, based on data from high-income, middle-income, and low-income countries (7). This highlights the global public health significance of birth defects, as well as significant regional differences in incidence, influenced by social, environmental, and biological factors. It is worth noting that for many severe congenital abnormalities, existing treatments are mostly palliative, and curing these defects is difficult (8, 9). Therefore, strengthening preventive interventions for birth defects has crucial public health implications.

The prevention strategies for birth defects are typically divided into three levels: primary prevention, secondary prevention, and tertiary prevention (10). Primary prevention focuses on reducing the risk of birth defects through preconception interventions, including health education, appropriate nutritional supplementation, preconception care, and genetic counseling (11). Secondary prevention is mainly carried out during pregnancy, involving prenatal screening and diagnosis techniques to identify high-risk fetuses early and implement interventions to reduce the incidence of birth defects (12). Tertiary prevention occurs in the neonatal period, focusing on early screening and timely treatment to minimize the clinical consequences of birth defects and improve the quality of life of affected children (13). In secondary prevention, prenatal screening plays a key role, aiming to identify high-risk fetuses early and providing a basis for subsequent prenatal diagnosis. Current major techniques for prenatal screening include imaging examinations (such as ultrasound), serological screening (e.g., Down syndrome screening), and molecular biology methods (e.g., non-invasive prenatal testing, NIPT), with a combined detection rate of up to 98.2% (14).

Despite significant progress in prenatal screening technologies, the diagnostic rate of prenatal screening in China remains relatively low, particularly among high-risk pregnant women (15). Notably, even when high-risk pregnant women receive enhanced prenatal care, this gap persists, as they often face barriers to completing confirmatory diagnostic procedures (e.g., amniocentesis). For instance, some women decline invasive diagnostic testing due to fears of miscarriage, financial constraints, or inadequate counseling. The popularity of prenatal diagnosis and its acceptance by pregnant women are influenced by various factors, including health literacy, the importance they place on prenatal screening, misconceptions about diagnostic risks, and concerns or fears about potential risks (16–18). Therefore, improving prenatal diagnosis rates among high-risk pregnant women through effective interventions that address these specific barriers is an urgent public health issue, as this population carries a substantially elevated baseline risk of fetal anomalies.

With the rapid development of mobile health technology (mHealth), digital health tools such as smartphone applications and WeChat platforms have gradually become important carriers of health interventions (19, 20). Studies have shown that mHealth technology has significant effects on improving maternal and child health. Ameyaw et al. confirmed through empirical research that mobile health applications effectively improve maternal nutrition and significantly reduce the risk of pregnancy complications (21). Atukunda and other scholars further discovered that providing personalized health information through SMS and audio messages and involving pregnant women’s social support networks (including family and friends) can significantly increase the utilization of maternal health services (22). In China, WeChat, with its convenience and wide user base, has become an important tool for maternal health management. This platform not only provides personalized health guidance to pregnant women but also promotes the acquisition and practical application of health knowledge through interactive online consultations and health education push notifications (23). WeChat is a multi-functional social platform developed by Tencent, with over 1 billion active users worldwide. It supports text, voice, and video messaging between individuals and allows users to create group chats with up to 500 members for large-scale interactions. Additionally, the “Moments” feature enables users to share updates, photos, and videos, similar to social media feeds. WeChat also supports Mini-Programs, which allow third-party developers to offer various services, including health consultations and online medical services. Based on this evidence, the present study believes that mobile health interventions, especially those using WeChat platforms, are highly suitable for increasing prenatal diagnosis rates among high-risk pregnant women, thereby improving their pregnancy outcomes. Although existing public health studies have confirmed that various interventions can increase the prenatal diagnosis rate among high-risk pregnant women, there is currently a lack of systematic research guided by health behavior theory models focusing on prenatal diagnosis rates (24). Therefore, this study aims to use the WeChat platform as a mobile health intervention tool guided by the Behavior Change Wheel theory and Family Health Theory, to improve adherence to prenatal diagnostic procedures among high-risk pregnant women, and enhancing their pregnancy outcomes.

**Figure 1:**
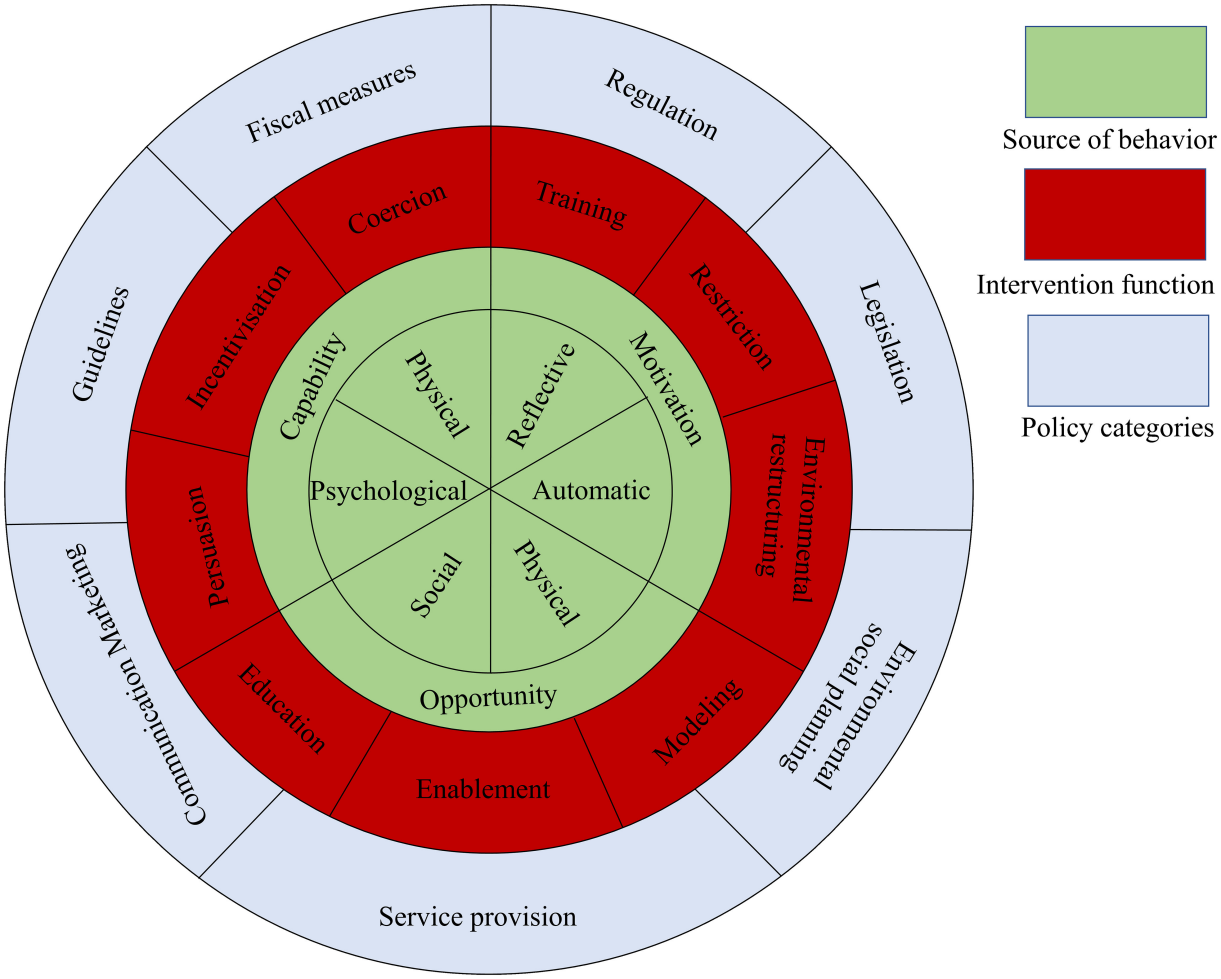
Behavior change wheel theory.

**Figure 2:**
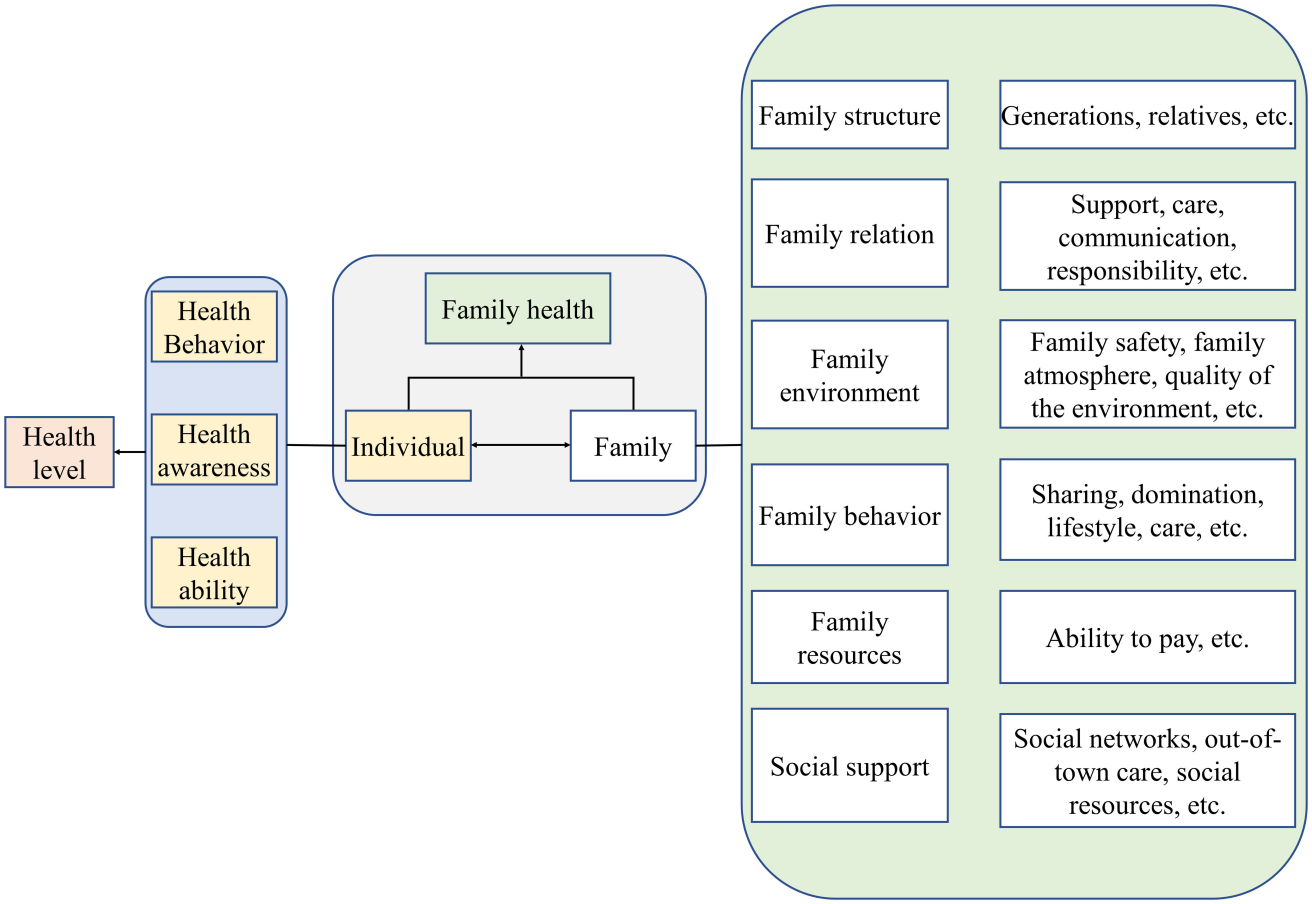
Conceptual framework of family health.

The Behavior Change Wheel (BCW), shown in **Figure 1**, was developed by Susan Michie and her research team in 2011 as a systematic framework for behavior change interventions (25). The theory integrates 19 related behavior change models and aims to help intervention designers understand why individuals fail to engage in specific behaviors, establish behavior goals, and select the core focus of interventions, providing a clear implementation path for systematic behavior change. Meanwhile, Sharon Denham innovatively introduced the concept of “family health,” which breaks the traditional focus on individual health in research (26). Based on this, Weiss-Laxer and other scholars further developed a more comprehensive conceptual framework for “family health” using the Delphi Method, as shown in **Figure 2** (27). They define “family health” as a comprehensive resource dependent on the family unit, derived from the health status, abilities, behavioral traits, personality, and internal interactions of family members, as well as the synergistic effects of external resources like physiological, social, emotional, economic, and medical support that the family can access. Therefore, combining the BCW and Family Health Theory provides a new theoretical perspective and practical approach for health behavior interventions, especially within the context of the family as a basic social unit. By integrating environmental factors from the BCW theory, the COM-B model (Capability, Opportunity, Motivation), and intervention functions, along with key elements from Family Health Theory, such as internal family interactions, family resource acquisition, and external family support, this approach offers a scientifically grounded and effective intervention plan for improving the overall health behaviors of individuals and families. Based on this, the current study will use the BCW and Family Health Theory as the theoretical foundation to develop a health education intervention model targeting high-risk pregnant women, aiming to improve their rate of prenatal screening and enhance pregnancy outcomes.

## 2 Methods

### 2.1 Design

**Figure 3:**
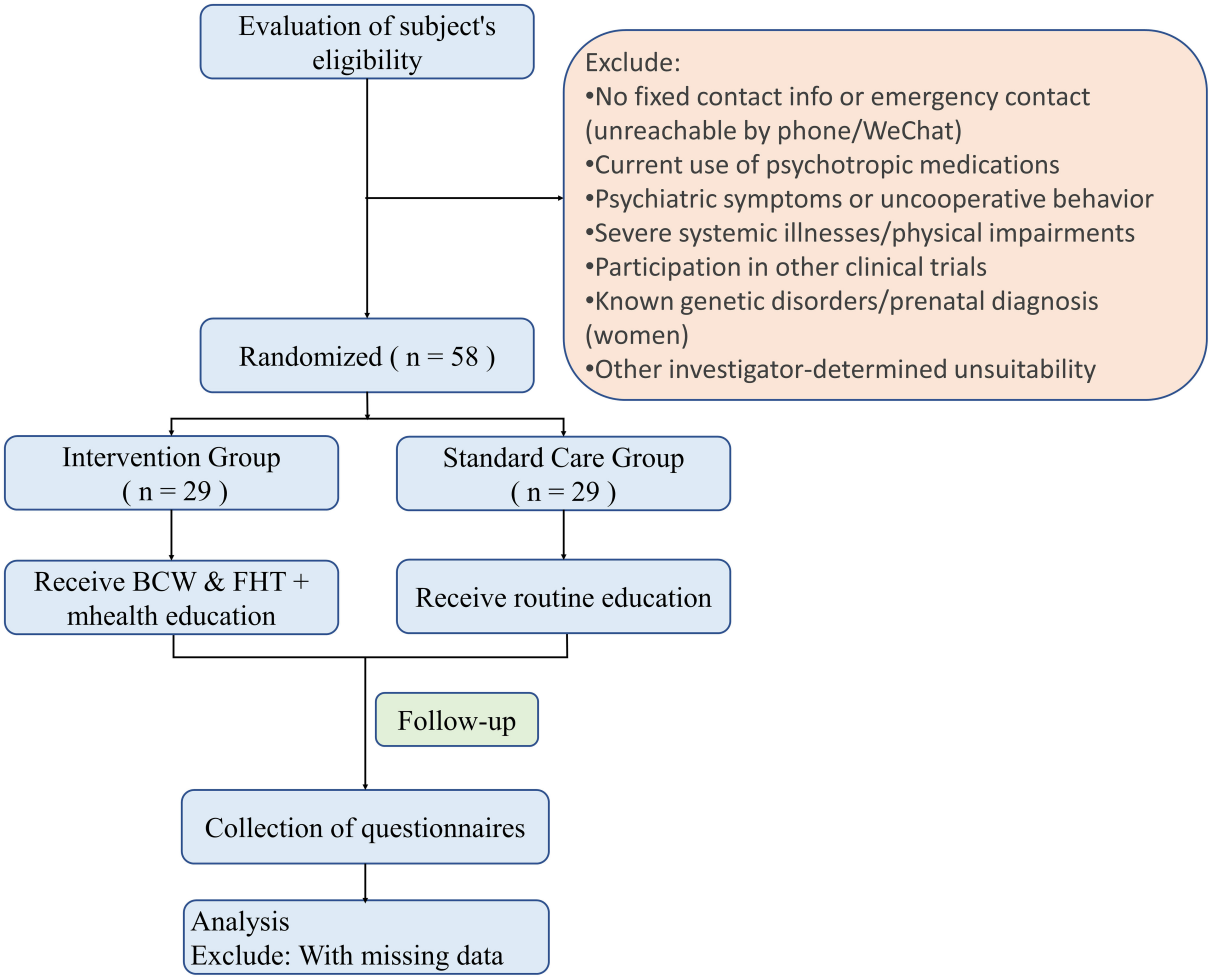
Flow chart of participants recruitment and study implement.

**Figure 4:**
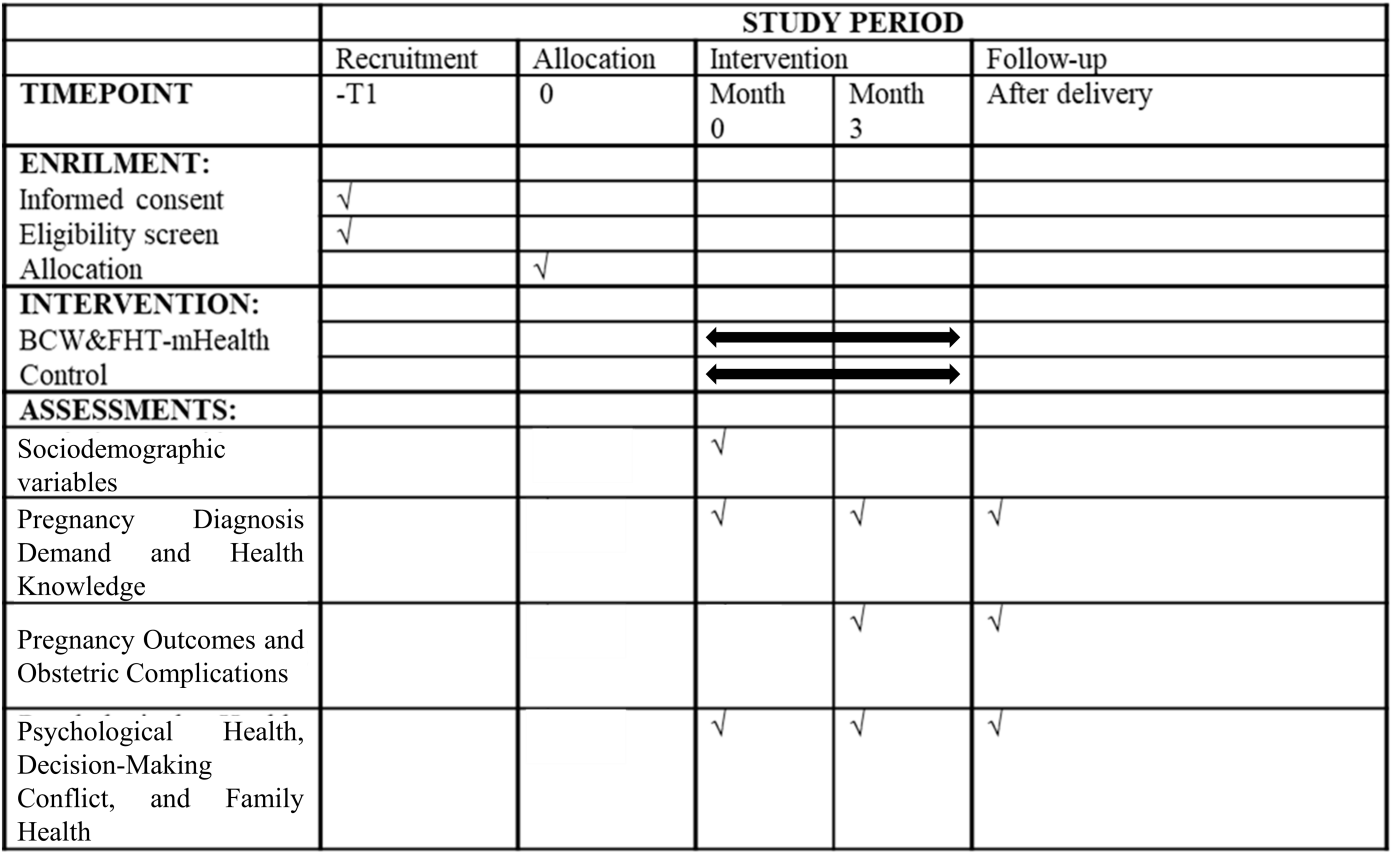
Timeline for study enrollment, intervention, and evaluation study setting and randomization.

This study is a single-blind, interventional randomized controlled trial conducted at the Inner Mongolia Maternity and Child Health Care Hospital. The aim is to evaluate the impact of a mobile health intervention based on the Behavior Change Wheel theory and Family Health Theory on the prenatal diagnosis rate and pregnancy outcomes of high-risk pregnant women undergoing prenatal screening. The study will be conducted from June 2025 to June 2026. The flowchart of the study is shown in **Figure 3** and **4**.

### 2.2 Participants

The participants in this study will be high-risk pregnant women undergoing prenatal screening at the Inner Mongolia Maternity and Child Health Care Hospital. Prior to enrollment, the research team will assess eligibility and have participants sign informed consent before randomly assigning them to either the intervention or standard care group, with 29 participants in each group, totaling 58 participants. The inclusion and exclusion criteria are as follows:

**Inclusion Criteria**

- Aged 18 years or older.
- High-risk pregnancy as determined by prenatal screening results.
- Basic ability to use mobile devices and WeChat.
- Willingness to participate and sign an informed consent form.

**Exclusion Criteria**

- No fixed contact information, no family member available for contact, or unable to be reached via phone or WeChat.
- Use of any psychiatric medications.
- Presence of mental symptoms such as unclear consciousness, speech difficulties, or lack of cooperation.
- Presence of severe systemic diseases or serious physical dysfunction.
- Participation in other clinical trials.
- Any other reasons deemed unsuitable for participation.
- Women who have been diagnosed with genetic diseases or have already undergone prenatal diagnosis.

### 2.3 Sample size estimation

This study is a randomized controlled trial, where the intervention group will receive the mobile health intervention and the standard care group will receive standard care. The outcome measure is the prenatal diagnosis demand. Based on previous randomized controlled trials related to willingness to undergo screening for related diseases, the intervention group’s demand proportion is 53.4%, and the standard care group’s is 34%, showing an increase of approximately 19.4% (28). We predict that the mobile health intervention based on the Behavior Change Wheel theory and Family Health Theory may lead to a doubling of the demand proportion (38.8%), reaching 72.8% (29). The expected demand proportion for the intervention group is 72.8%, and for the standard care group is 34%. Using a two-sided test with α = 0.05, β = 0.2, and power (1-β) = 80%, the sample size ratio between the two groups is 1:1. After using the PASS software (version 2021) to calculate the relevant values, it was determined that 23 participants per group would be required. Considering a 20% dropout rate, the final sample size for each group is 29 participants, for a total of 58 participants.

### 2.4 Randomization and blinding

**Figure 5:**
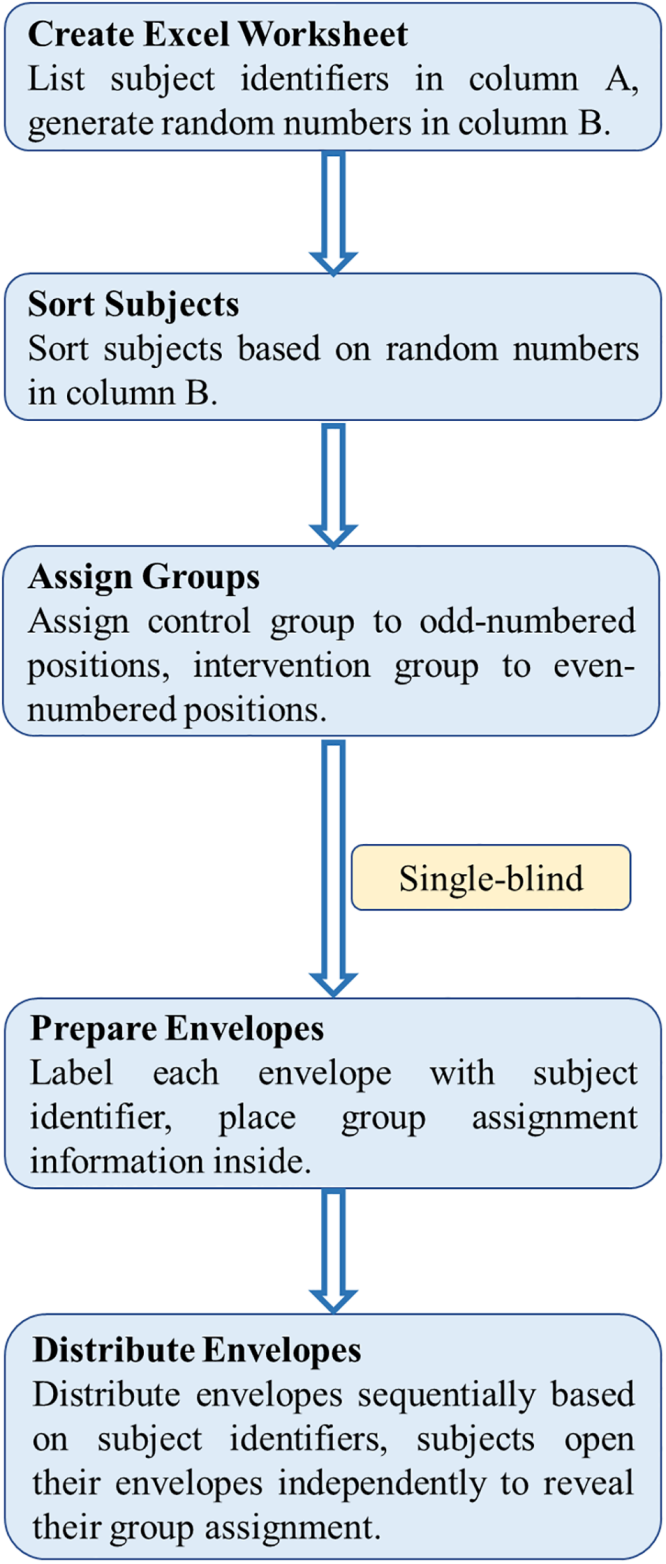
The process of randomization.

In this study, randomization was conducted using Excel software to ensure unbiased group allocation (**Figure 5**). The procedure is as follows: First, a new worksheet was created in Excel, with numbers from 1 to N in column A (where N is the total number of subjects), representing the subject identifiers. In column B, the formula =RAND() was used to generate a random number between 0 and 1 for each subject. Then, to prevent any changes to the random numbers, they were copied and pasted as “values.” After that, the subjects were sorted based on the random numbers in column B, and group allocations were made according to the sorted order.

During group allocation, the order after sorting determines each subject’s group. Specifically, subjects in odd-numbered positions in the sorted order were allocated to the standard care group, while those in even-numbered positions were allocated to the intervention group. It should be noted that the subject identifiers remain in column A, but the actual group assignment is based on the sorted order in column B.

To ensure blinding, a single-blind design was adopted, where subjects were unaware of which group they were assigned to, but the evaluators were not blinded. To implement allocation concealment, the envelope method was used for group allocation. The procedure is as follows: Sufficient opaque envelopes were prepared, each marked with a unique identifier number corresponding to the subject’s identifier in column A of the Excel sheet. A small slip of paper with the group allocation information (Intervention Group and Standard Care Group) was placed inside each envelope. This allocation information corresponds to the envelope number, which matches the subject’s identifier in column A, ensuring that the envelope received by each subject corresponds to their assigned group based on the randomization process. The envelopes were then sealed.

After enrollment, subjects received an envelope in sequential order based on their subject identifier in column A. Each subject opened their envelope independently to reveal their group assignment. The distribution and opening of the envelopes were supervised by a designated person, and the process was recorded. All unused envelopes were securely stored to maintain the integrity of allocation concealment and the single-blind design.

### 2.5 Theory framework

#### 2.5.1 Behavior change wheel theory

The Behavior Change Wheel theory consists of three levels:

1. **The inner level (source of behavior):** This level focuses on the COM-B model (Capacity, Opportunity, Motivation - Behavior), which identifies the factors that influence the behavior of individuals and helps in recognizing potential behavioral barriers. It aims to explore the sources of target behavior and the underlying causes driving it.
2. **The middle level (intervention function)**: This layer develops nine distinct intervention functions, such as education, restriction, and incentivization, which are designed to encourage the formation of the target behavior. These functions can be tailored to the research environment, aiding in the creation of interventions that promote the desired behavior.
3. **The outer level (policy categories)**: This level addresses the external factors influencing the interventions, such as laws, regulations, financial incentives, guidelines, communication, service provision, and environmental or social planning. These policy areas assist in shaping the interventions and ensuring the stability of the behavior over time.

The three levels interact and are interrelated, working together to drive the establishment of healthy behaviors in patients for better intervention outcomes. The process of designing an intervention based on this theory typically involves: understanding the behavior (defining the existing problem with behavior-related terminology, selecting the target behavior, describing the behavior in detail, and identifying what can be changed), determining intervention options (choosing intervention functions and policy types), and specifying the content and delivery methods (selecting behavior change techniques and intervention delivery modes).

#### 2.5.2 Family health theory

Family health transcends the mere aggregation of individual health statuses and extends beyond traditional definitions confined by blood relationships. It encompasses multidimensional aspects including family structure, interpersonal dynamics, living environments, behavioral patterns, and internal resource allocation. Critically, family health also incorporates external social support systems, particularly access to social networks, healthcare resources, and other societal assets. These intrinsic and extrinsic factors interact reciprocally with individuals, permeating daily life to modify health-related behaviors, awareness, and competencies—thereby collectively shaping the family’s health trajectory. Simultaneously, individual behaviors, perceptions, and capacities reconfigure the relational framework, environmental contexts, and collective actions of the family unit.

The construct of family health emerges through the synergistic interplay between internal family frameworks and externally accessible resources. This paradigm synthesizes key elements from established concepts such as family structure, functional capacity, and social connectivity, while enhancing a family’s ability to mobilize external resources and strengthen societal engagement. By emphasizing health-relevant determinants, it establishes an integrative nexus between individual well-being and population health outcomes. Such conceptualization provides a robust foundation for investigating health disparities and designing family-centric interventions within public health research.

#### 2.5.3 Combination of behavior change wheel theory and family health theory

The convergence of the Behavior Change Wheel (BCW) framework and Family Health Theory offers a transformative paradigm for designing health behavior interventions, particularly salient in contexts where the family unit constitutes the fundamental societal building block. By integrating the BCW’s emphasis on environmental determinants, the COM-B model (Capability, Opportunity, Motivation-Behavior), and targeted intervention functions with Family Health Theory’s focus on intrafamilial interactions, resource mobilization, and external support systems, this synthesis facilitates the development of multidimensional intervention strategies. Such strategies holistically address both individual-level behavioral drivers and family-level systemic dynamics, thereby optimizing health behavior modification across micro and macro units of analysis.

This interdisciplinary approach enables the creation of context-sensitive interventions that leverage familial cohesion as a catalyst for sustainable behavioral change. Specifically, the BCW’s systematic mapping of behavior change techniques aligns with Family Health Theory’s emphasis on resource accessibility and social embeddedness, allowing researchers to operationalize interventions that simultaneously strengthen family health capital and enhance individual COM-B components. The resultant framework provides a novel theoretical scaffold for investigating how family-mediated interventions can amplify the efficacy of traditional behavior change models while addressing health disparities rooted in familial and social contexts.

### 2.6 Intervention

This study established a multidisciplinary team consisting of genetic experts, nursing specialists, genetic doctors, and public health experts. Through several rounds of in-depth discussions, team members combined their expertise to develop comprehensive intervention strategies aimed at addressing the behavioral barriers to prenatal diagnosis participation among high-risk pregnant women, incorporating mobile health technology as a key tool.

#### 2.6.1 Intervention group

**Figure 6:**
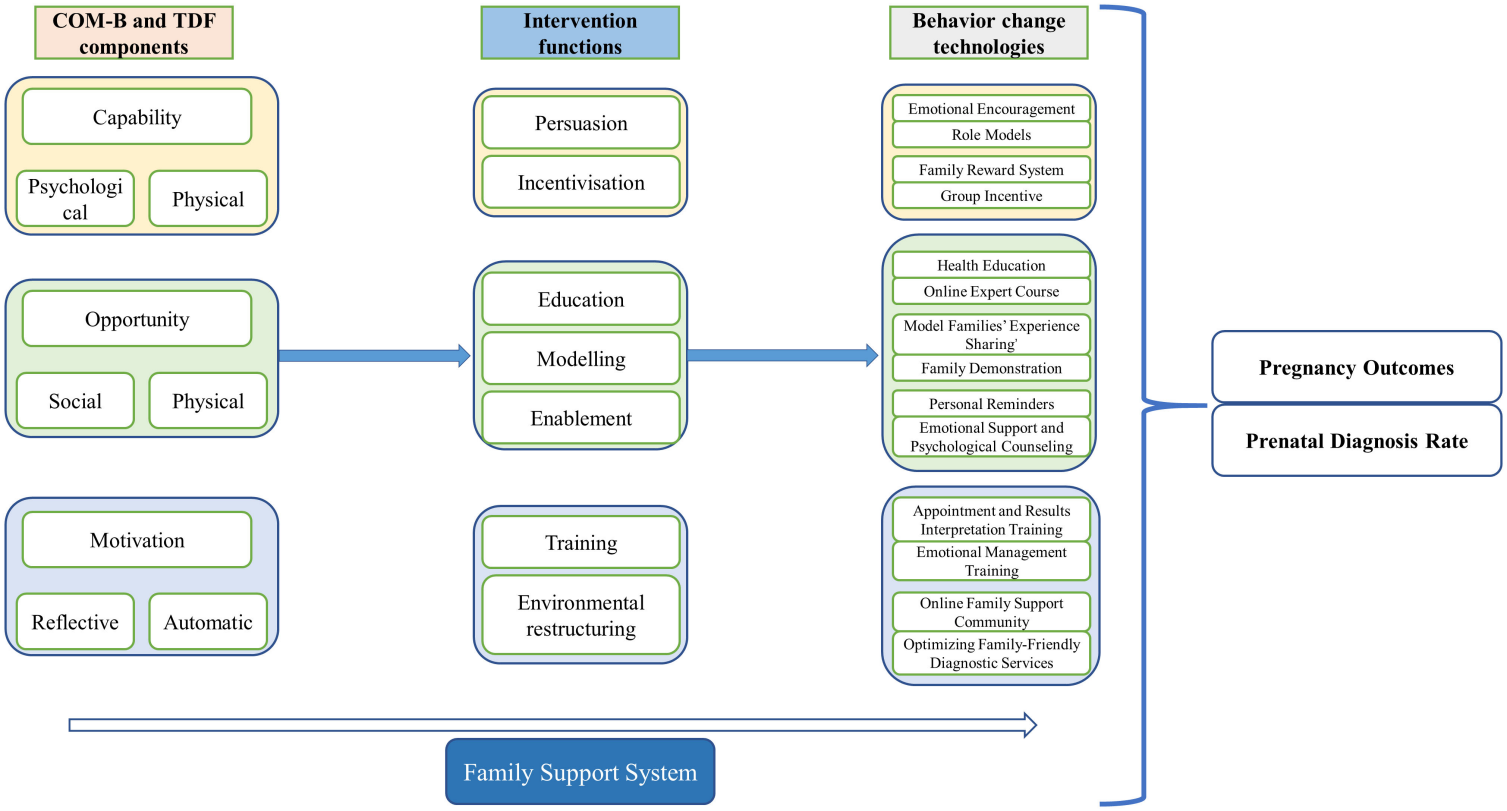
Combination of behavior change wheel theory and family health theory.

The intervention program combines the Behavior Change Wheel (BCW) theory and Family Health Theory, with a particular emphasis on helping high-risk pregnant women overcome negative emotions such as anxiety and depression through family support systems, ultimately improving pregnancy outcomes and increasing prenatal diagnosis rates (**Figure 6**). The intervention will last for 12 weeks, during which the WeChat platform will be used consistently to deliver continuous health education, emotional support, and behavioral feedback. The goal is to help pregnant women overcome emotional barriers and increase their participation in prenatal diagnosis and improve their pregnancy outcomes through emotional management, behavioral support, and family involvement.

**Step 1: Understanding behavioral issues (based on COM-B model and family health integration analysis)**

Using the BCW's Capability, Opportunity, Motivation-Behavior (COM-B) model and integrating family health factors, this step will thoroughly analyze the behavioral barriers to high-risk pregnant women participating in prenatal diagnosis.

- **Target behavior:** The target behavior is to enhance the awareness of high-risk pregnant women and their families regarding prenatal diagnosis, promote joint participation by the pregnant women and their partners, and help families correctly interpret the diagnosis results. Additionally, emotional management and psychological support will be provided to reduce anxiety and depression, improving pregnancy outcomes.
- **Behavioral barrier analysis (integrating family health factors):**
  - **Capability:**
    ▪ **Psychological capability:** High-risk pregnant women and their family members (especially husbands) often have limited knowledge about prenatal diagnosis, its processes, and its importance. Cognitive gaps within the family may lead to inconsistent decisions or lack of support. Providing easy-to-understand educational materials for families and emphasizing joint participation in health decision-making can address these gaps. Additionally, high-risk pregnant women often experience anxiety and depression, which can affect their decision-making and behavior. Emotional support and psychological interventions will help manage these emotions and increase their participation in prenatal diagnosis.
    ▪ **Physical capability:** Pregnant women’s physical conditions, such as pregnancy symptoms, anxiety, or concerns, may affect their participation in diagnosis. Providing emotional comfort and emotional support from family members can help alleviate these issues. Support from husbands and other family members can enhance the pregnant women’s willingness to participate in and complete the diagnosis.
  - **Opportunity:**
    ▪ **Physical opportunity:** Access to medical resources in the family’s area, convenience of prenatal diagnosis services, and reasonable diagnostic costs all impact a woman’s opportunity to participate in diagnosis. The intervention will ensure that families have easy access to diagnostic appointments and information. Additionally, providing family-based diagnostic education will help reduce fear and uncertainty about prenatal diagnosis, boosting confidence in participating.
    ▪ **Social opportunity:** Family health theory emphasizes the importance of family support systems. The attitudes and support of family members (particularly husbands, parents, or in-laws) are crucial for a pregnant woman’s decision to undergo prenatal diagnosis. If family members lack knowledge or awareness of the importance of diagnosis, it may affect the woman’s decision. Therefore, the intervention will strengthen communication and interaction among family members, providing knowledge dissemination at the family level. By strengthening the role of family members and providing emotional support, anxiety and depression can be reduced.
  - **Motivation:**
    ▪ **Reflective motivation:** The attitude and awareness of the target family regarding prenatal diagnosis directly affect behavior. Pregnant women and their families may feel anxious about the diagnosis results, fearing the detection of genetic diseases or abnormal outcomes. Family-level health education will help reduce misunderstandings and anxiety, enhancing the understanding of the necessity and benefits of prenatal diagnosis.
    ▪ **Automatic motivation:** Habits, emotions, and family culture can have a significant impact on behavior. Some families may have doubts or resistance towards medical diagnosis. By leveraging emotional support, emotional encouragement, and sharing successful case studies, families can be effectively motivated to actively participate in the diagnosis process.

**Step 2: Choosing intervention functions and strategies (BCT) (integrating family health factors)**

Based on the integration of the COM-B model and Family Health Theory, the following intervention strategies will help overcome behavioral barriers and focus on emotional management, ultimately increasing prenatal diagnosis rates and improving pregnancy outcomes:

- Education:
  - Purpose: Improve the understanding of prenatal diagnosis for pregnant women and their family members, and provide psychological health education to help them understand the sources of anxiety and depression and how to cope.
  - Intervention Strategies:
    ▪ Health education: Design family health education materials for high-risk pregnant women and their partners, providing information on prenatal diagnosis, diagnostic procedures, and interpreting results (e.g., via WeChat messages, video courses). Design and push psychological health education materials to help identify early signs of anxiety and depression and offer emotional regulation techniques.
    ▪ Online expert courses: Online Expert Courses will be delivered via WeChat platform. Invite genetic experts to hold online lectures for families, answering questions related to the diagnosis process and boosting confidence in prenatal diagnosis.
- Persuasion:
  - Purpose: Use emotional encouragement and positive feedback to reduce anxiety and worry, and increase positive attitudes towards prenatal diagnosis.
  - Intervention Strategies:
    ▪ Emotional encouragement: Share case stories related to family health, emphasizing the importance of prenatal diagnosis for family health. By showing successful diagnosis experiences of real families, families will view diagnosis as an act of caring for their loved ones, eliminating the fear of diagnosis results.
    ▪ Role models: Invite families who have completed prenatal diagnosis to share their experiences and encourage partners and family members to talk about the support they provided during the diagnosis process. This can influence other families to form positive health behaviors and increase trust in prenatal diagnosis, reducing anxiety about negative results.
- **Incentivisation**:
  - Purpose: Encourage families to actively participate in prenatal diagnosis through a reward system while alleviating the negative impact of emotional barriers on decision-making.
  - Intervention strategies:
    ▪ Family Reward System: Set up a family health reward program to encourage both partners to participate in prenatal diagnosis. Families who complete the diagnosis will be rewarded or recognized with a “Healthy Family” title, acknowledging their active participation in health decision-making.
    ▪ Group incentives: Organize health knowledge competitions, family check-in activities, and emotional regulation challenges in WeChat groups to encourage family members to participate and share their diagnostic process, strengthening collective involvement and family cohesion.
- **Training**:
  - Purpose: Enhance the self-management abilities of pregnant women and their family members during the prenatal diagnosis process and provide emotional management tools.
  - Intervention strategies:
    ▪ Appointment and results interpretation training: Create a detailed prenatal diagnosis appointment guide and provide hands-on training for family members to ensure they can complete the diagnostic process smoothly. Provide online courses on interpreting diagnostic results, helping families understand the meaning of results and subsequent measures.
    ▪ Emotional management training: Organize emotional management training to teach how to cope with prenatal anxiety and depression, helping pregnant women adjust their mindset.
- **Environmental restructuring:**
  - Purpose: Create a supportive social environment for the target families, increasing their access to diagnostic services and knowledge, as well as emotional support.
  - Intervention strategies:
    ▪ Online family support community: Establish a family health support platform through WeChat groups, encouraging family members to share experiences, discuss diagnostic-related issues, and provide online Q&A with doctors. This promotes mutual support within families and communities, reduces feelings of isolation, and enhances emotional support.
    ▪ Optimizing family-friendly diagnostic services: Provide convenient diagnostic appointment services, design diagnostic reminders, and support functions (e.g., pre- and post-consultation calls).
- **Modeling:**
  - Purpose: Encourage target families to model healthy behaviors through examples.
  - Intervention strategies:
    ▪ Model Families’ Experience Sharing: Push stories of families who have completed prenatal diagnosis and overcame anxiety with emotional support. Show how they successfully managed their emotions and actively participated in the diagnosis process. Real-life case examples will motivate other families to get involved and reduce their fears and anxieties about the process.
    ▪ Family demonstration: Encourage families, especially husbands, who have completed the prenatal diagnosis to share how they provided emotional support during the diagnosis process and helped pregnant women overcome emotional barriers. These model families’ experiences will serve as learning examples for other families.
- **Enablement:**
  - Purpose: Help the target families overcome behavioral barriers and take action immediately by providing personalized support and real-time reminders, especially to help pregnant women and family members manage emotions and participate in prenatal diagnosis.
  - Intervention strategies:
    ▪ Personalized reminders: Send customized reminders in the WeChat group for each family, reminding them of the prenatal diagnosis appointment and important details. Also, remind pregnant women and their family members to focus on emotional regulation. For example: "Dear XX family, your prenatal diagnosis appointment is coming up, please make sure you're ready and maintain a positive attitude!"
    ▪ Emotional support and psychological counseling: Provide psychological counseling for women and family members who are worried about the diagnosis results or feel anxious about the process. This will help them overcome emotional barriers and complete the prenatal diagnosis with peace of mind.

**Step 3: Phase-wise implementation and evaluation**

**implementation in phases:**

The intervention will be implemented in three phases, with each phase involving specific tasks, staff responsibilities, and procedures to ensure smooth execution.

**Phase 1: Dissemination of information and emotional support (Week 1-4)**

- **Responsible personnel**: A team consisting of 3 trained nurses, 2 psychologists, and 1 genetic expert will handle the educational and emotional support activities.
- **Competencies**: Nurses will be trained in prenatal care and communication skills, psychologists will specialize in emotional support and anxiety management during pregnancy, and the genetic expert will have expertise in prenatal diagnostics and genetic counseling.
- **Process**: The team will use WeChat groups and official WeChat accounts to deliver educational content on prenatal diagnosis, covering topics such as its importance, process, and potential outcomes. Psychological support will also be provided to help reduce anxiety and fear among pregnant women. Each session will last approximately 30 minutes, with weekly check-ins to track progress. Additionally, the genetic expert will host online lectures for families, answering questions related to the diagnostic process and boosting confidence in prenatal diagnosis. Success stories from previous participants will also be shared in the early educational materials to further enhance participants’ confidence.
- **Incident management**: If a participant shows severe anxiety or depressive symptoms beyond normal concerns, a psychologist will be assigned for an in-depth individual consultation to provide additional emotional support.

**Phase 2: Appointment scheduling and family support (Week 5-8)**

- **Responsible personnel**: A team consisting of 2 nurses, 1 social worker, 1 psychologist, and 1 genetic expert.
- **Competencies**: The nurses will guide participants in scheduling their prenatal diagnosis appointments, the social worker will facilitate family communication and ensure family members understand their supportive roles, the psychologist will provide emotional support and counseling to help manage anxiety and emotional concerns, and the genetic expert will provide specialized input on prenatal diagnosis and genetic counseling.
- **Process**: During this phase, the nurses will assist pregnant women and their families in scheduling prenatal diagnosis appointments and provide support to ensure they understand the entire process. The social worker will educate family members on how to emotionally and physically support the pregnant woman. The genetic expert will host online lectures for the families to answer questions about the diagnostic process and boost their confidence in prenatal diagnosis. Each session will last 45 minutes and will be scheduled bi-weekly. The social worker will also send customized reminders to each family in the WeChat group, reminding them of the upcoming prenatal diagnosis appointment and key details while encouraging emotional regulation, such as, “Dear XX family, your prenatal diagnosis appointment is approaching, please be prepared and maintain a positive mindset!” For those who are anxious about the results or the process, the psychologist will provide counseling to help them manage their emotional concerns and complete the prenatal diagnosis with peace of mind.
- **Incident management**: If a participant experiences logistical issues, such as scheduling conflicts or transportation problems, the social worker will step in to provide solutions or connect the participant with local resources to resolve these issues.

**Phase 3: Reinforcement and role modeling (Week 9-12)**

- **Responsible personnel**: The team will include 1 nurse, 1 psychologist, and 1 program coordinator.
- **Competencies**: The nurse and psychologist will provide continued health education and emotional support, while the program coordinator will be in charge of managing the group activities and reinforcing family participation.
- **Process**: In the final phase, the nurses and psychologist will reinforce the support behaviors of family members by sharing success stories from families who have completed the process. The nurses will also offer incentives to those who successfully complete the prenatal diagnosis. The program coordinator will organize group activities such as knowledge quizzes and family challenges to encourage participation. Each session will last 60 minutes and will be conducted weekly.
- **Incident management**: If a participant experiences a setback, such as resistance from family members or emotional distress, the psychologist will provide additional counseling, and the nurse will assist with medical information or clarification. The program coordinator will facilitate family discussions to ensure everyone is on the same page and help resolve any disagreements or concerns.

**Evaluation and feedback:**

- Regular evaluations will be conducted to track changes in the target families’ knowledge levels, prenatal diagnosis participation rates, and anxiety/depression levels. Feedback will be provided to adjust the intervention strategies in a timely manner.

This integrated intervention model, combining the Behavior Change Wheel theory and Family Health Theory, effectively leverages the power of the family to encourage high-risk pregnant women to participate more actively in prenatal diagnosis, providing support from the whole family and improving emotional wellbeing, ultimately leading to better pregnancy outcomes.

#### 2.6.2 Standard care group

The standard care group will receive routine prenatal care without the additional mobile health intervention. Routine care includes basic information provided by medical staff at regular checkups, such as prenatal vitamins, ultrasound scans, and general prenatal education.

### 2.7 Collection and screening of health education materials

#### 2.7.1 Sources of health education materials

The health education materials used in this study will be collected from a variety of domestic and international platforms to ensure comprehensive and diverse content. These sources include well-known online platforms such as WeChat, Wikipedia, medical Q&A forums like “Doctor’s Q&A,” and specialized databases like ZhiNET. The collection will not be limited to text-based content but will also include video and graphic materials, which can better engage different learning preferences and facilitate a more accessible educational experience for the target audience.

All materials chosen for this study will be scientifically accurate and derived from credible sources. Specifically, medical documents and educational resources will be gathered from trustworthy online platforms, such as Baidu documents, authoritative medical websites, and peer-reviewed literature. Proper citations and references will be provided for all included materials to maintain academic integrity. Each selected piece of content will be evaluated for its factual correctness, reliability, and relevance to the study’s focus on birth defects, ensuring it provides up-to-date and valuable information to the public.

#### 2.7.2 Screening of health education materials

After the collection and organization of the health education materials, a thorough screening process will be conducted. A panel of experts specializing in the field of birth defects will be responsible for reviewing the content of all materials. These experts, with deep knowledge in prenatal care, genetic counseling, and related areas, will evaluate the materials for scientific accuracy, clarity, and their ability to effectively communicate essential information to patients and the general public.

The experts will suggest any necessary modifications to improve the materials, ensuring they are scientifically sound and easily understood by a wide range of audiences. Any materials that do not meet the established criteria will be excluded from the final selection. The remaining high-quality materials will then be categorized into relevant areas such as awareness of birth defects, prevention strategies, prenatal care, and genetic counseling, among others. This classification will allow for the creation of targeted, informative push recommendations tailored to the specific needs of patients and the public.

The final selection of health education materials will be compiled into a comprehensive database, which will serve as a central resource for the study. This database will include key details for each material, such as the title, relevant links, sources, and material types (e.g., articles, videos, infographics). This resource will also be used to generate personalized push notifications to patients, offering them the most relevant and up-to-date educational materials. By ensuring that only high-quality, evidence-based resources are included, this process will support effective health education and help patients make informed decisions regarding birth defect prevention and care.

### 2.8 Outcome measures

There is a total of nine outcomes in the study. Medical staff will use the Questionnaire Star web platform to distribute the questionnaire, and patients will complete the questionnaire online and be instructed by medical staff either in person (at the time of hospitalisation) or by telephone (after discharge). Patient demographic information will be collected through the case system and self-designed questionnaires. (**Table 1**)

**Table 1:** Data collection components and collection timeline.

Type of research	Data collection component	Content	Timepoint	Month 3	After delivery
			Month 0		
Quantitative research	Sociodemographic variables	Sex, age, insurance status, place of residence, average monthly household income, educational level, parents’ education level, area of domicile,	✓		
	Demand for prenatal diagnosis	Willingness for diagnosis, plans for prenatal diagnosis within six months, or having undergone diagnosis	✓	✓	✓
	Pregnancy outcomes	Full-term birth, preterm birth, miscarriage, induced labor due to social factors, and induced labor due to chromosomal abnormalities	✓	✓	✓
	Health Knowledge and Birth Defect Prevention Knowledge Awareness	Self-designed questionnaire	✓	✓	✓
	Obstetric Complications	Gestational hypertension, postpartum hemorrhage, and low-quality infants.	✓	✓	✓
	Depression and anxiety	Patient Health Questionnaire-4 (PHQ-4)	✓	✓	✓
	Decisional Conflict	Decisional Conflict Scale (DCS)	✓	✓	✓
	Self efficacy	Decision Self-Efficacy Scale	✓	✓	✓
	Family Health	Family Health Scale-Short Form (FHS-SF)	✓	✓	✓
	Satisfaction	Decision-making participation satisfaction scale		✓	✓

#### 2.8.1 Primary outcomes

The primary endpoints are the demand for prenatal diagnosis and pregnancy outcomes. Demand is defined as the perceived or expressed need, typically associated with changes in health status. Demand is measured at the individual level both before and after the intervention. A participant is considered to have a screening demand if they answer “yes” to at least one of the following three questions: willingness for diagnosis, plans for prenatal diagnosis within six months, or having undergone diagnosis. The overall demand is the union of these three items. For participants who report having been diagnosed, the researchers will verify the diagnosis by reviewing relevant documents or appointment cards.

Pregnancy outcomes include full-term birth, preterm birth, miscarriage, induced labor due to social factors, and induced labor due to chromosomal abnormalities.

#### 2.8.2 Secondary outcomes

**Health knowledge and birth defect prevention knowledge awareness**

The awareness of health knowledge and birth defect prevention knowledge is measured through a self-designed questionnaire. Participants are asked to respond to questions about their understanding of relevant health knowledge and preventive measures, thereby assessing their level of awareness.

**Obstetric complications**

In this study, participants were asked to indicate whether they experienced the following obstetric complications: gestational hypertension, postpartum hemorrhage, and low-quality infants. Each complication was self-reported by the participants to provide a comprehensive understanding of their childbirth experience.

**Patient health questionnaire-4, PHQ-4**

The Patient Health Questionnaire-4 (PHQ-4) is a concise instrument developed to evaluate two primary dimensions of mental health: anxiety and depression. It consists of four items, the first two entries are designed to assess depressed mood, while the last two are used to assess anxiety, each rated on a 4-point Likert scale, with response options ranging from 0 (not at all) to 3 (nearly every day). Participants are instructed to consider their experiences of anxiety and depression over a specified time frame. The cumulative score ranges from 0 to 12, with elevated scores indicating increased symptom severity. This scale is user-friendly and facilitates healthcare providers in promptly identifying individuals potentially experiencing anxiety or depression, thereby enabling rapid initial assessments. The PHQ-4 has demonstrated robust internal consistency, evidenced by a Cronbach’s alpha of 0.82, highlighting its reliability for clinical application (30).

**Decisional conflict dcale, DCS**

The Decisional Conflict Scale (DCS), originally developed by O’Connor et al., is a widely acknowledged instrument for evaluating the extent of decisional conflict experienced by patients (31). In 2017, a team of Chinese researchers, including Li Yu et al., undertook the translation of the scale into Chinese, with the translated version achieving a Cronbach’s alpha of 0.897, indicating high internal consistency (32). The Chinese version of the DCS consists of 16 items, categorized into three principal dimensions: Information and Values (items 1–6), Decision Support and Decision Effectiveness (items 7, 9–11, 13–16), and Decision Uncertainty (items 8, 12). The scale employs a 100-point scoring system, where lower scores denote reduced decisional conflict. A score of 25 or higher suggests the presence of decisional conflict, while scores exceeding 37.5 may indicate potential delays in the patient’s decision-making process.

**Decision self-efficacy scale**

The Decision Self-Efficacy Scale, developed by Canadian nursing scholar O’Connor, is designed to evaluate an individual’s confidence in making informed healthcare decisions (33). The Chinese version, translated by Wang Sitong et al., was specifically applied to patients with primary liver cancer (34). This scale includes 11 items within a single dimension, each scored on a 5-point Likert scale ranging from “very unconfident” to “very confident”. The overall score is calculated by summing the individual item scores, averaging them (dividing by 11), and then multiplying by 25, resulting in a total score range from 0 to 100. Higher scores reflect greater decision self-efficacy. Studies have demonstrated the scale’s strong reliability and validity, with a content validity index of 0.966 and a Cronbach’s alpha of 0.918.

**Family health scale-short form, FHS-SF**

The Family Health Scale-Short Form (FHS-SF), developed by Crandall et al. (2020) is designed to assess family health (35). For our study, we used the Chinese version of the FHS-SF, which was translated by Wang et al. (2022). They demonstrated that this version possesses strong internal consistency and excellent test-retest reliability, confirming its validity as a reliable tool for evaluating family health (36). The scale includes four dimensions and a total of 10 items: family/social and emotional health processes, family healthy lifestyles, family health resources, and external social support. Participants rated each item on a five-point scale, ranging from “strongly disagree” to “strongly agree”. For three items related to “family health resources”, which were negatively worded, scores were reversed. The final Family Health Score is derived by summing the responses, with higher scores indicating better family health, and the total score ranging from 10 to 50.

**Decision-making participation satisfaction scale**

The Decision-Making Participation Satisfaction Scale, created by Xu Xiaolin, evaluates satisfaction with involvement in decision-making. It consists of 16 items across four dimensions: Information (4 items), Communication and Consultation (4 items), Decision-Making (3 items), and Overall Satisfaction and Confidence (5 items) (37). Participants rate each item using a 5-point Likert scale, with responses ranging from “very dissatisfied” to “very satisfied”. The total score, which ranges from 16 to 80, is calculated by summing the ratings, with higher scores reflecting greater satisfaction with decision-making participation. The scale has a Cronbach’s alpha of 0.899.

### 2.9 Data analysis

This study uses IBM SPSS Statistics 24.0 software for quantitative analysis to ensure the accuracy and reliability of the statistical data. In terms of descriptive statistics, we present the baseline characteristics of the participants, with continuous variables being summarized by means and standard deviations or medians and interquartile ranges, while categorical variables are presented as frequencies and percentages. This statistical approach effectively summarizes the basic information of the sample, providing a solid foundation for subsequent analysis. The distribution of continuous data will be tested for normality. Normally distributed data will be presented as mean ± standard deviation (M ± SD), and non-normally distributed data as median (IQR). The Mann–Whitney U test will be used to compare changes in scale scores between baseline and endpoint between the two groups, while the Wilcoxon test will compare differences within each group. The chi-square test will compare the demographic characteristics of the two groups. Statistical significance will be set at p < 0.05, using a two-sided test.

In the case of missing data, assumed to be missing at random, baseline variables will be used as auxiliary variables, and missing data will be imputed using multiple imputation methods. Linear regression and logistic regression will be used for analysis, and the final combined analysis will complete the imputation process.

### 2.10 Data management

#### 2.10.1 Data collection

All clinical and laboratory data will be carefully recorded in each subject’s medical record and Case Report Form (CRF). The CRF will be the main document for capturing study information, ensuring standardized data organization. The investigator is responsible for verifying the accuracy and completeness of the CRF, cross-checking it with source documents. Each CRF entry will be dated and signed to authenticate the data. A copy of the completed CRF will be submitted to the Principal Investigator (PI), while the original will remain with the investigator. Regular audits will be conducted to ensure data consistency and resolve any discrepancies.

#### 2.10.2 Storage and archiving of data

After the trial, all data, including subject IDs, source data, CRFs, and investigator documents, will be securely archived in a designated database, following institutional and regulatory guidelines. The data will be protected from unauthorized access and stored in physical or digital formats, with backups to prevent loss. The data will be retained for a minimum period, in compliance with legal and ethical standards. After the retention period, the data will be securely disposed of, ensuring confidentiality and adherence to data protection laws.

### 2.11 Clinical supervision

To ensure trial quality and integrity, clinical supervision will be implemented according to ethical and legal standards, with a focus on ICH-GCP guidelines. Supervision will involve randomized monitoring visits and ongoing oversight, following IZKS Standard Operating Procedures (SOPs). Clinical exams and trial procedures will be observed during in-person visits, with supervisors reviewing the Case Report Forms (CRFs) and cross-checking data against source documents to ensure accuracy. Any discrepancies will be promptly addressed. The investigator will facilitate monitoring by providing access to all necessary study documentation, including CRFs, medical records, and laboratory results, and will support the monitors throughout their visits to ensure efficient data collection. Through strict oversight, the trial will prioritize participant safety, maintain data integrity, and ensure that study objectives are met accurately. Supervisors will offer feedback and corrective actions as needed, helping to identify issues early and ensuring the success of the trial. Regular monitoring and transparent documentation will contribute to the study’s overall validity and reliability.

## 3 Discussion

This study aims to assess the impact of mobile health education, grounded in the Behavior Change Wheel theory and Family Health Theory, on the participation rate in prenatal diagnosis and pregnancy outcomes among high-risk pregnant women. To our knowledge, this is the first study to use the Behavior Change Wheel theory and Family Health Theory as the theoretical framework for health management interventions targeting high-risk pregnant women in prenatal screening. High-risk pregnant women are more likely to face pregnancy complications, making improving prenatal diagnosis participation and pregnancy outcomes a key issue in global public health. While some studies suggest that behavioral interventions can effectively improve maternal health behaviors, mobile health education interventions targeting high-risk pregnant women remain limited, especially those combining the of Behavior Change Wheel theory with Family Health Theory.

This study’s innovation lies in integrating its theoretical framework with intervention strategies. The Behavior Change Wheel theory offers a multidimensional framework to help high-risk pregnant women identify and overcome cognitive and emotional barriers to prenatal diagnosis, thereby enhancing their self-efficacy in health behaviors. Family Health Theory highlights the crucial role of family members in managing the health of high-risk pregnant women, with research demonstrating that family support is vital in promoting maternal health behaviors. By integrating these two theories, this study aims to offer a comprehensive support system for high-risk pregnant women, assisting them in understanding and engaging in prenatal diagnosis while also fostering health management during pregnancy.

Another key feature of this study is the use of mobile health education interventions. As mobile health technology advances, personalized intervention methods have emerged as a new trend in public health. Mobile health interventions provide convenient and flexible health management options, particularly for the busy high-risk pregnant population, through real-time health feedback and tailored suggestions. Furthermore, mobile health education is cost-effective and scalable, making it an effective, low-cost intervention for improving prenatal diagnosis participation and pregnancy outcomes among high-risk pregnant women.

This study employs a randomized controlled trial design to assess the effects of personalized mobile health education interventions on prenatal diagnosis participation rates and pregnancy outcomes among high-risk pregnant women. By collecting baseline data from participants, including sociodemographic information and health knowledge, the study aims to deepen our understanding of the factors influencing intervention effectiveness. Based on these data, future intervention strategies could be optimized by tailoring content to better meet the needs of individual participants, thus improving intervention effectiveness and enhancing compliance.

Despite the innovative and practical significance of this study, several limitations require further exploration and resolution in future research. First, the intervention targets only high-risk pregnant women participating in prenatal screening from the Inner Mongolia Maternity and Child Health Care Hospital, with the study sample being limited, which may introduce regional and selection biases. Second, the study primarily relies on self-reported data, which may introduce reporting bias. Additionally, while the study designs an intervention model grounded in the Behavior Change Wheel theory and Family Health Theory, the evaluation of its effectiveness may still be influenced by various factors. For instance, the socio-economic status, cultural background, and family support of the pregnant women may all influence the intervention outcomes. Furthermore, future studies should explore the generalizability of such interventions to low-risk populations, where prenatal diagnosis rates may be constrained by distinct mechanisms.

## 4 Conclusion

This study is expected to provide important practical evidence for promoting prenatal diagnosis among the high-risk pregnant women group. If the intervention is effective, this mobile health education model based on the Behavior Change Wheel theory and Family Health Theory will offer medical professionals a new intervention design concept. Furthermore, future research could further expand the intervention content and methods, exploring how emerging technologies (such as big data analysis and artificial intelligence) can be integrated to provide more personalized and precise health guidance for pregnant women.
